# Supplementary material for: RADAR: annotation and prioritization of variants in the post-transcriptional regulome of RNA-binding proteins
Source: Genome Biol. 2020 Jul 30;21:151. doi: 10.1186/s13059-020-01979-4 (PMC7391703; doi:10.1186/s13059-020-01979-4)
Supplement: Supplementary file 2 — Additional file 2: Supplemental Figures and Tables for RADAR providing more information on results and methods. [file 13059_2020_1979_MOESM2_ESM.pdf]

Additional File 2

"RADAR: annotation and prioritization of variants in the post-transcriptional regulome for RNA-binding proteins"

Table of Contents

1 Defining the RBP regulome using eCLIP data 3
1.1 Functional Annotation of RBPs 3
1.2 Functional Annotation of RBP binding sites 4
2 Inference of structural conservation on eCLIP peaks 5
3 RBP binding network analysis 5
3.1 RBP network hub analysis 5
3.2 RBP co-binding analysis 8
4 RBP-gene association by RBP KD experiments 8
5 Highlighting key regulators through expression profiles 9
6 Simple flowchart of the RADAR scoring system 10
7 Applying RADAR to pathological germline variants 11
8 Comparison of RADAR to other methods 12
9 Applying RADAR to somatic variants in cancer 12
10 Relative Importance of RADAR Features 14
11 Using cell type specific information as validation 15
12 Comparison of CLIP methods 16
12.1 Comparison of eCLIP data to other RBP binding data 16
13 Cross-population and species conservation and GC bias 17
13.1 Inference of cross-population conservation of RBP binding sites 17
13.2 Inference of cross-species conservation of RBP binding sites 19
14 Motif analysis 19
14.1 Motifs from RNA Bind-n-Seq experiments 19
14.2 Motifs from de novo discovery 21
14.3 Motif disruption calculation using MotifTools 22
15 Downloading and Using the RADAR software 22
16 References 24

Additional File 2: Supplementary Figures

Figure S 1 Annotation summary of RBPs 3
Figure S 2. Increased cross-population conservation after added Evofold feature to RBP peaks.. 5
Figure S 3. Distribution of binding RBP numbers.. 6
Figure S 4 Adjusted rare variant percentage vs. number of RBPs binding in coding regions. Regions with top 5% and 1% of RBPs binding are defined as the hot and ultra-hot regions. 7
Figure S 5 Adjusted rare variant percentage vs. number of RBPs binding in noncoding regions. Regions with top 5% and 1% of RBPs binding are defined as the hot and ultra-hot regions. 7
Figure S 6 Co-binding analysis of RBPs 8
Figure S 7. Schematic of highlighting variants that breaks gene-RBP association from RBP knockdown experiments. 9
Figure S 8. Details about RADAR scoring system.. 10
Figure S 9. Baseline RADAR scores of HGMD vs. all 1kG variants 11
Figure S 10 RADAR score in somatic variants 12
Figure S 11 Highlighted breast cancer variants in the 3' UTR region 13
Figure S 12 Flowchart of Random forest analyses for RADAR variable importance................. 14
Figure S 13. RADAR score comparison in tissue-specific and commonly expressed genes by separating K562 and HepG2 data 15
Figure S 14 RADAR score comparison in liver cancer specific and common tumor driver genes by separating K562 and HepG2 data 16
Figure S 15 Number of Peaks for Various RBP CLIP methods 16
Figure S 16. Background Rare variant percentage vs. GC 17
Figure S 17 Rare variant enrichment after GC correction in coding and noncoding regions respectively. The dashed blue/red line is the genome average without GC correction for coding and noncoding regions, and the solid blue/red line is the background after GC correction. Blue/Red star on top of each bar indicate significantly enriched in rare variants after GC correction in one-sided binominal test against the coding/noncoding average. 18

Additional File 2: Supplementary Tables

Table S 1 Specific RBPs and their functions. 3
Table S 2 RBP binding peaks within annotated regions.. 4
Table S 3 Comparison of RADAR to Funseq2.. 12
Table S 4 Summary of AUC values from leave-one-out analysis 14
Table S 5 List of RBPs that have both eCLIP and Bind-n-Seq experiments............................... 19

1 Defining the RBP regulome using eCLIP data

1.1 ***Functional Annotation of RBPs***

eCLIP is an enhanced version of the crosslinking and immunoprecipitation (CLIP) assay, and is used to identify the binding sites of RNA binding proteins (RBPs). We collected all available eCLIP experiments from the ENCODE data portal (encodeprojects.org). There were 178 experiments from K562 and 140 experiments from HepG2, totaling 318 eCLIP experiments from all available ENCODE cell lines (released and processed by July 2017).

These experiments targeted 112 unique RBP profiles. eCLIP data was processed by ENCODE 3 uniform data processing pipeline. The eCLIP peak calling method and processing pipeline were developed by Gene Yeo's lab at the University of California, San Diego (https://github.com/YeoLab/clipper, CLIP-seq cluster-identification algorithm[1]). For each peak, the enrichment significance was calculated against a paired input, and we filtered those peaks with a flag of 1000, which are considered to be the statistically significant peaks.

We summarized the list of available RBPs in Table S 1(in separate data package) and provided detailed annotation as we can. We also summarized different categories of RBPs as well as the relative size of the RBP regulome in Figure S 1.

Figure S 1 Annotation summary of RBPs

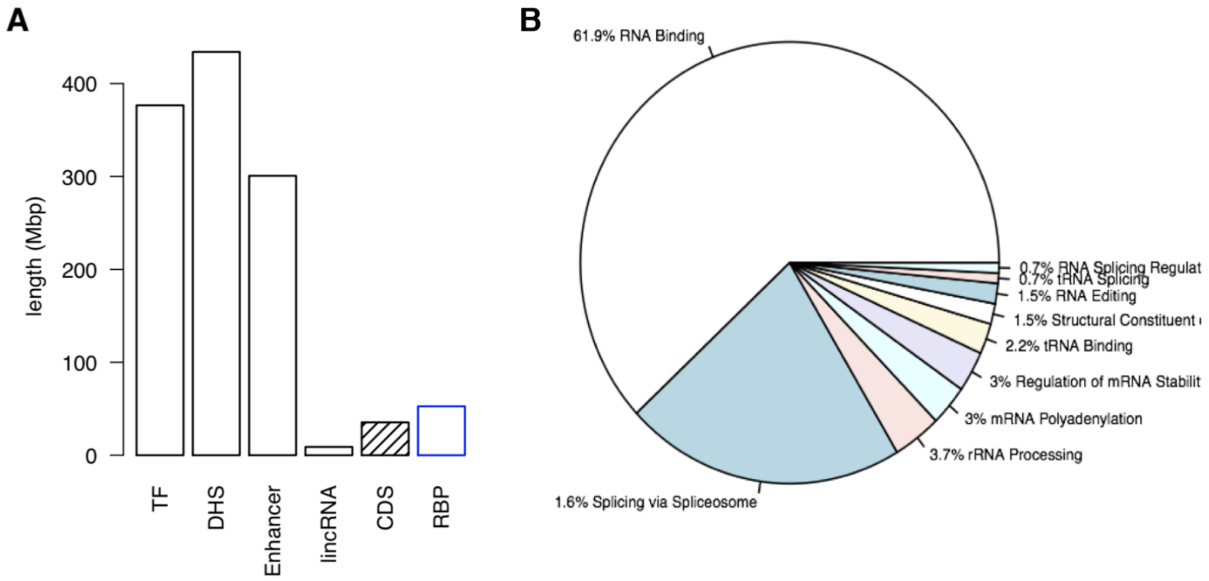

Below is a table categorizing each RBP by their function, many of which are splicing related. We also include a downloadable link on our website of eCLIP data annotated by each RBPs specific function, which can easily be filtered for splicing related RBPs, and found at http://radar.gersteinlab.org/splicing.zip.

Table S 1 Specific RBPs and their functions.

Category	RBPs
RNA Binding	DDX3X, DDX59, DGCR8, DROSHA, EWSR1, HNRNPA1, HNRNPC, HNRNPK, HNRNPM, HNRNPU, HNRNPUL1, IGF2BP3, ILF3, KHDRBS1, NONO, NPM1, PCBP2, PRPF8, PTBP1, RBFOX2, RBM15, RBM22, SAFB2, SF3A3, SRSF7, SRSF9, TAF15, TARDBP, TNRC6A, U2AF1, U2AF2, AARS, AUH, CPSF6, CSTF2, CSTF2T, DDX24, DDX42, DDX55, DDX6, DHX30, DKC1, EIF4G2, FAM120A, FASTKD2, FMR1, FUBP3, FXR1, FXR2, GEMIN5, GRSF1, IGF2BP1, IGF2BP2, KHSRP, LARP4, LARP7, LIN28B, LSM11, MTPAP, NOL12, NSUN2, PPIL4, PUM2, PUS1, QKI, RBM27, RPS11, RPS5, SERBP1, SF3B4, SFPQ, SLBP, SLTM, SMNDC1, SRSF1, SUGP2, SUPV3L1, TIA1, TRA2A, TROVE2, UPF1, XPO5, ZRANB2
tRNA Binding	AARS, NSUN2, XPO5
tRNA Splicing	CSTF2
Pre mRNA Splicing	GTF2F1, HNRNPA1, HNRNPC, HNRNPK, HNRNPM, HNRNPU, HNRNPUL1, NONO, PCBP2, PRPF8, PTBP1, RBM15, RBM22, SF3A3, SRSF7, SRSF9, U2AF1, U2AF2, BUD13, CDC40, CSTF2, EFTUD2, GEMIN5, GPKOW, NCBP2, SF3B1, SF3B4, SRSF1, TRA2A
Splicing Regulation	RBFOX2
Polyadenylation	CPSF6, CSTF2, GRSF1, MTPAP
mRNA Stability	FMR1, KHSRP, PUM2, SERBP1
rRNA Processing	DKC1, RPS11, RPS5, SBDS, XRN2
Ribosome Structure	RPS11, RPS5
RNA Editing	DKC1, PUS1

1.2 Functional Annotation of RBP binding sites

From the raw peaks from ENCODE, we further removed the ones overlapped with either blacklist regions from ENCODE (https://www.encodeproject.org/annotations/ENCSR636HFF/, select hg19) or gap regions like Telomere and Centromere from UCSC (ftp://hgdownload.cse.ucsc.edu/goldenPath/hg19/database/gap.txt.gz). In total, over 99% of the binding locations are preserved after blacklist removal.

We further tried to annotate these peak regions by dividing them into different annotation categories from Gencode V19. Specifically, we extracted 7 different annotation categories, including coding exons, 3'UTR, 5'UTR, 3'UTR extended (1000bp downstream), 5'UTR extended (1000bp upstream), nearby intron (up to 100bp to the exon/intron junctions), and deep introns. For any region that might overlap two annotation categories, we only preserve one in the order mentioned above. The raw number of nucleotides in each annotation category is given in Table S 2.

Table S 2 RBP binding peaks within annotated regions

Annotation Type	Nucleotides
**Coding Exon**	156069

**3’ UTR**	65447
**5’ UTR**	28339
**3’ UTR extended**	39985
**5’ UTR extended**	45036
**Nearby Intron**	102892
**Deep Intron**	312424

2 Inference of structural conservation on eCLIP peaks

We downloaded the Evofold bed files for hg19 from UCSC Genome Browser and used it as a feature for our analysis. Specifically, we found that after requiring that any RBP peaks should also be with conserved structure in Evofold, these binding sites significantly increases its population-level conservations (as shown in Figure S 2).

Figure S 2. Increased cross-population conservation after added Evofold feature to RBP peaks

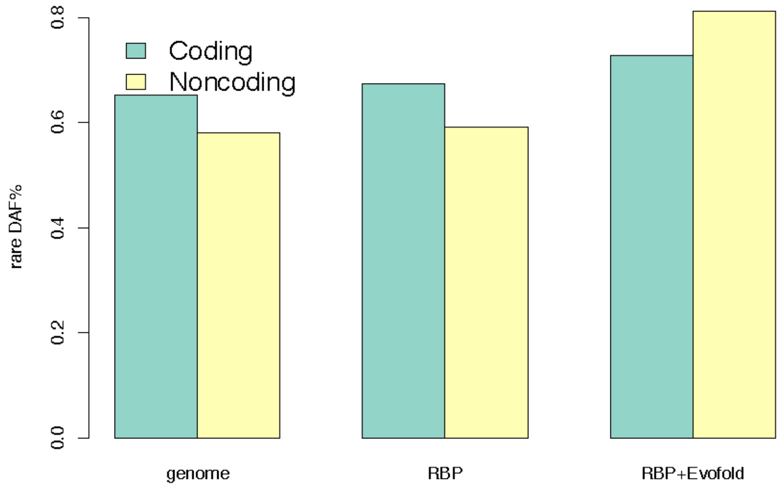

3 RBP binding network analysis

3.1 ***RBP network hub analysis***

We also inferred the RBP binding hubs and hypothesized that they are under higher negative selection since once mutated, there is a higher chance to alter RBP regulations. Specifically, we calculated the number of RBPs that bind to each nucleotide and the distribution is given in Figure

S 3. As expected, due to the specificity of RBP binding events, the majority (over 60%) of the RBP regulome was surrounded by only one RBP.

Figure S 3. Distribution of binding RBP numbers.

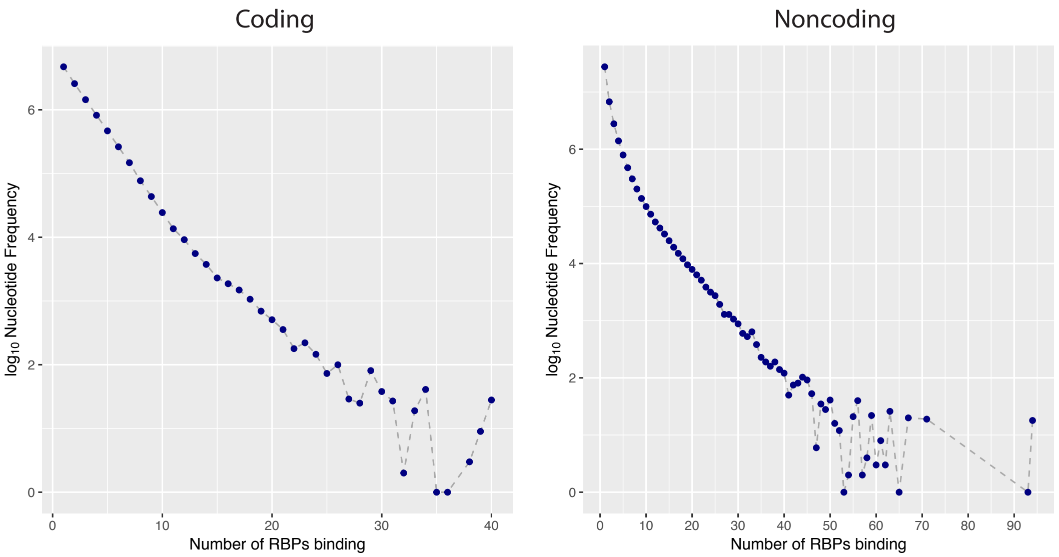

We then calculated the enrichment of rare variants for regions with at least 1, 2, 3, …, 112 RBPs. We corrected the GC bias in a similar way to section 13.1. As expected, as the number of RBPs increased, we observed an obvious trend of enrichment of rare variants (Figure S 4 and Figure S 5). For instance, in the noncoding region, around 5% of the regulome is surrounded with at least 5 RBPs, and they exhibited 3% more rare variants compared to the whole genome. For regions that are surrounded by at least 10 RBPs, which are around 1% of the whole regulome, we observed up to 12% more rare variants (Figure S 5). This observation significantly supports our hypothesis that the RNA regulome hubs are under stronger purifying selection, and should be given higher priority when evaluating the functional impacts of mutations.

Figure S 4 Adjusted rare variant percentage vs. number of RBPs binding in coding regions. Regions with top 5% and 1% of RBPs binding are defined as the hot and ultra-hot regions.

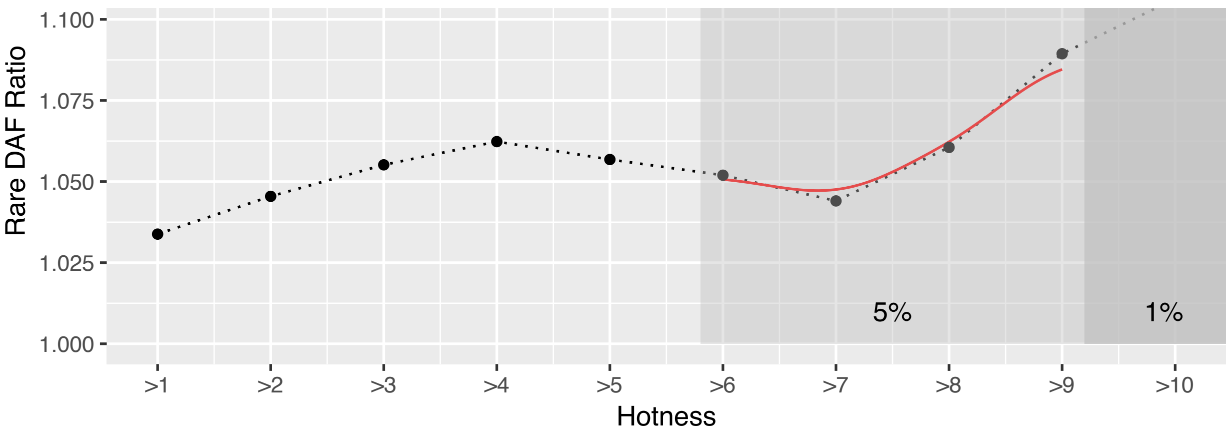

Figure S 5 Adjusted rare variant percentage vs. number of RBPs binding in noncoding regions. Regions with top 5% and 1% of RBPs binding are defined as the hot and ultra-hot regions.

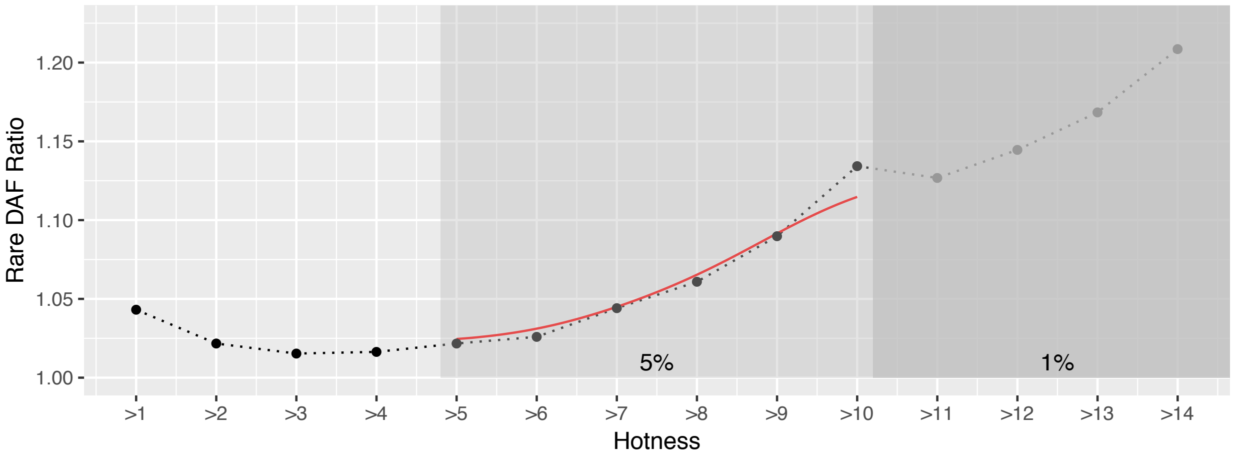

We also investigated the RBP binding events interactions from two aspects: co-binding analysis and RBP binding hub analysis. Details are given in the following sections.

3.2 RBP co-binding analysis

Figure S 6 Co-binding analysis of RBPs

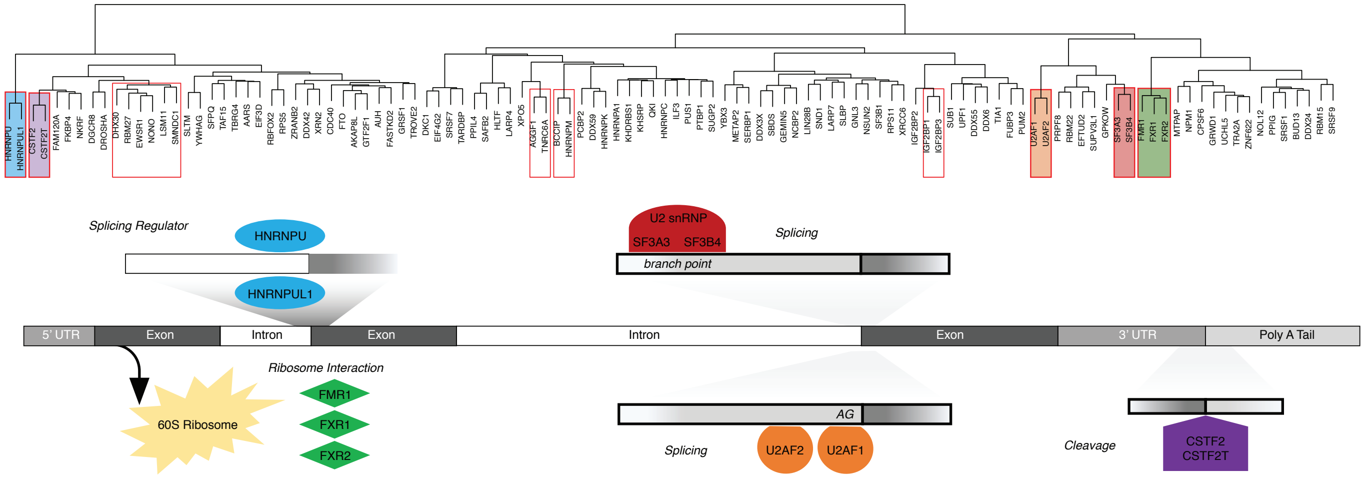

We defined the co-binding percent of each RBP pair by the ratio of overlapping nucleotides over the union of nucleotides in their binding peaks. Then we constructed a co-binding percentage matrix for all RBPs to measure their co-binding status. Then, we performed a hierarchical clustering of this matrix using the pvrect function from the pvclust package in R with an alpha value of 0.02 to identify the co-binding pairs. The resulting clusters of RBPs with significance were found to follow patterns of functional co-binding found in literature and results are given in Figure S 6.

Using a significance level threshold of 0.05, we found several pairs of well-known regulatory partners with different binding preferences. For example, the famous heterogeneous nuclear ribonucleoprotein (hnRNP) family protein HNRNPU and its paralog HNRNPUL1 were found to bind together in the nearby intron region, probably regulating the pre-mRNA splicing process[2]. F3A3 and SF3B4, which encode two units of splicing factor 3a protein, were also found to co-bind in the nearby intron region in our data[3, 4]. The SR family protein U2AF1 and U2AF2 are found to co-bind near the intron/exon junctions to jointly control splicing events[4, 5]. Two cleavage stimulation factor (CSTF) complex proteins, CSTF2 and CSTF2T, were found to bind near the 3' UTR, and were reported to be associated with 3' end cleavage and polyadenylation of pre-mRNAs. Consistent with previous report, three functional similar genes FMR1, FXR1, and FXR2 were found to co-express, and shuttle between the nucleus and cytoplasm and associate with polyribosomes, predominantly with the 60S ribosomal subunit[6, 7]. The discovery of the co-binding of such functional relevant proteins at various regions indicates the high quality of our regulome.

4 RBP-gene association by RBP KD experiments

RNA-seq expression profiling before and after shRNA mediated RBP depletion from ENCODE can help infer the gene expression changes introduced by RBP knockdown. Variants

with disruptive effect on RBP binding may affect or even completely remove RBP binding and hence affect gene expressions in a similar way. A schematic of our procedure is given in Figure S 7.

Specifically, we first collected 472 shRNA RNA-seq experiments (supplementary table S5) and extracted the differentially expressed genes (supplementary table S6) from such experiments. For example, in Figure S 7, we define the G1-RBP2 association from the RBP knockdown experiment. Then within the extended G1 region, we extracted all motif breaking variant effect for all possible RBPs (within peaks). If any variant breaks RBPs that has an association with G1, we give it an extra credit in our baseline score.

Figure S 7. Schematic of highlighting variants that breaks gene-RBP association from RBP knockdown experiments.

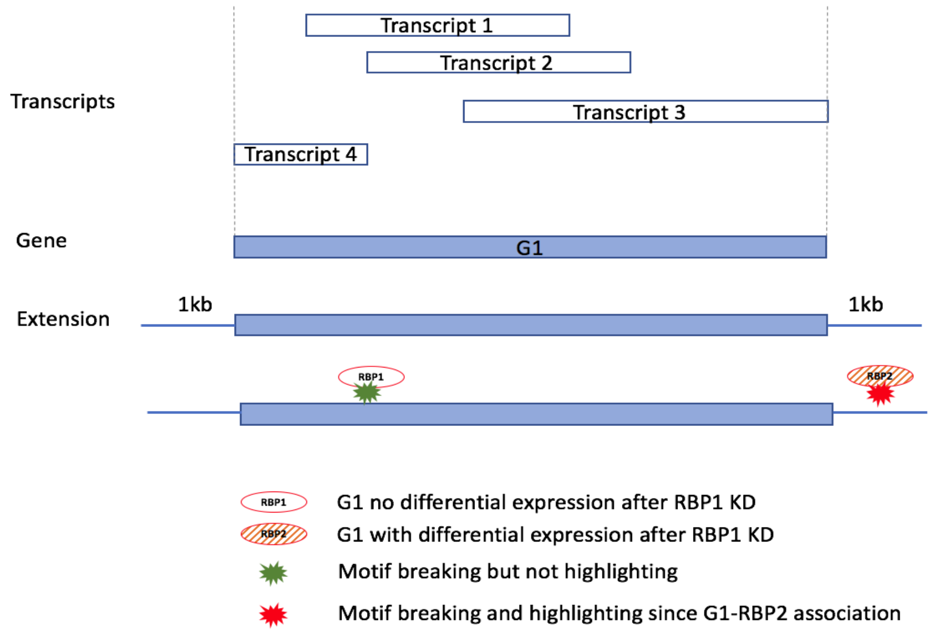

5 Highlighting key regulators through expression profiles

In order to detect the key RBP regulators that drive the disease-specific gene expression patterns, we constructed RBP regulatory networks and incorporated gene expression profiles to find RBPs that are associated with expression changes in patient cohorts.

Specifically, we first downloaded the full set of TCGA expression profiles for 24 cancer types. In order to get a robust differential expression analysis, we excluded 6 cancer types that have less than 10 normal expression profiles. For each cancer type, both tumor and normal expression were given to DESeq2[8] to identify tumor-specific gene differential expression status.

Then we tried to set up RBP regulatory network directly from the RBP peaks. We used the full set of protein coding genes in Gencode v19, and then extracted their 3'UTR regions. For any

protein coding gene, a RBP is supposed to regulate this gene if this RBP has a binding peak intersecting the 3'UTR region.

We inferred the regulation power of each RBP by through a regression approach of the above differential expression status and RBP network connectivity. We used the absolute value of regression coefficient as the aggregated RBP regulation power. The full table of regulation powers in all 19 cancer types were given in Additional file 1: T5. Interestingly, we found that for the RBPs with larger regulation power are those tends to be known to associated with cancer, as listed in Additional file 1: T5.

For RBPs with high regulation powers, we also performed a patient-wise regulation power inference, where the differential expression is determined as the individual expression fold change. Then, we tried to use such individual regulatory power to predict disease prognosis. We downloaded the patient survival data from TCGA and performed survival analysis using the survival package in R (version 2.4.1-3).

6 Simple flowchart of the RADAR scoring system

To better illustrate how RADAR works, we provided a simple flowchart of calculations for scoring a variant using RADAR in Figure S 8.

Figure S 8. Details about RADAR scoring system

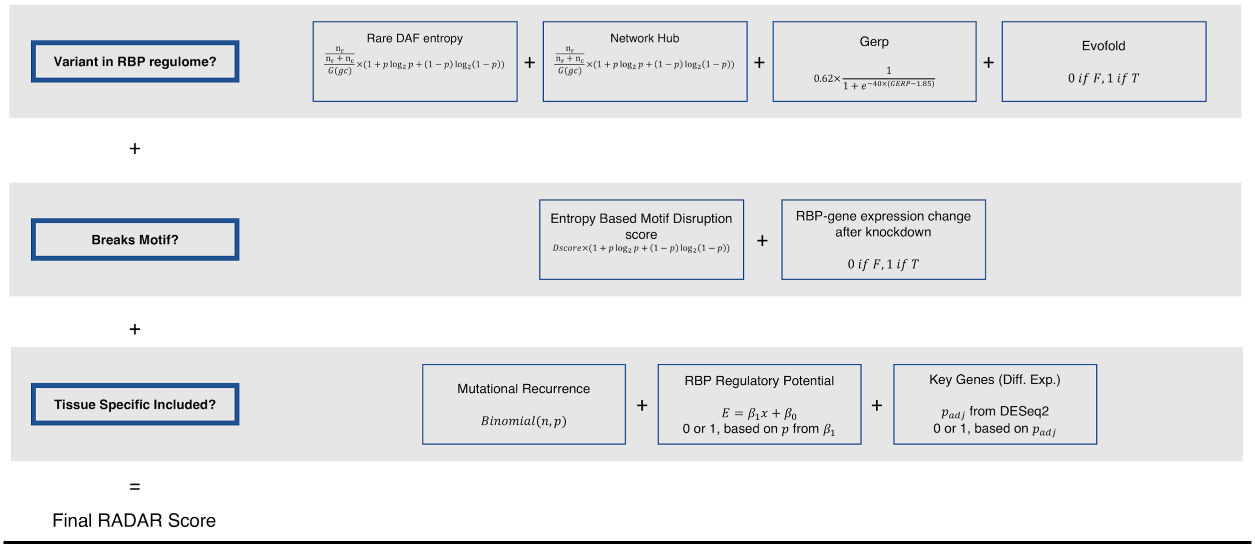

7 Applying RADAR to pathological germline variants

HGMD variants (version 2015) were used in our analysis. For Figure 6, the signal tracks for the eCLIP experiments were directly downloaded from ENCODE. Funseq2 and CADD scores were directly calculated from their website. The list of highly prioritized variants discovered only by RADAR were provided in Supplementary Table S9. The comparison of RADAR baseline scores of HGMD vs 1kg variants were given in Figure S 9. Since the majority of 1kG variants are located far away from the exon regions, we further extracted variants that are only inside the RBP regulome for both HGMD and 1kG variants and compared their RADAR baseline scores.

Figure S 9. Baseline RADAR scores of HGMD vs. all 1kG variants

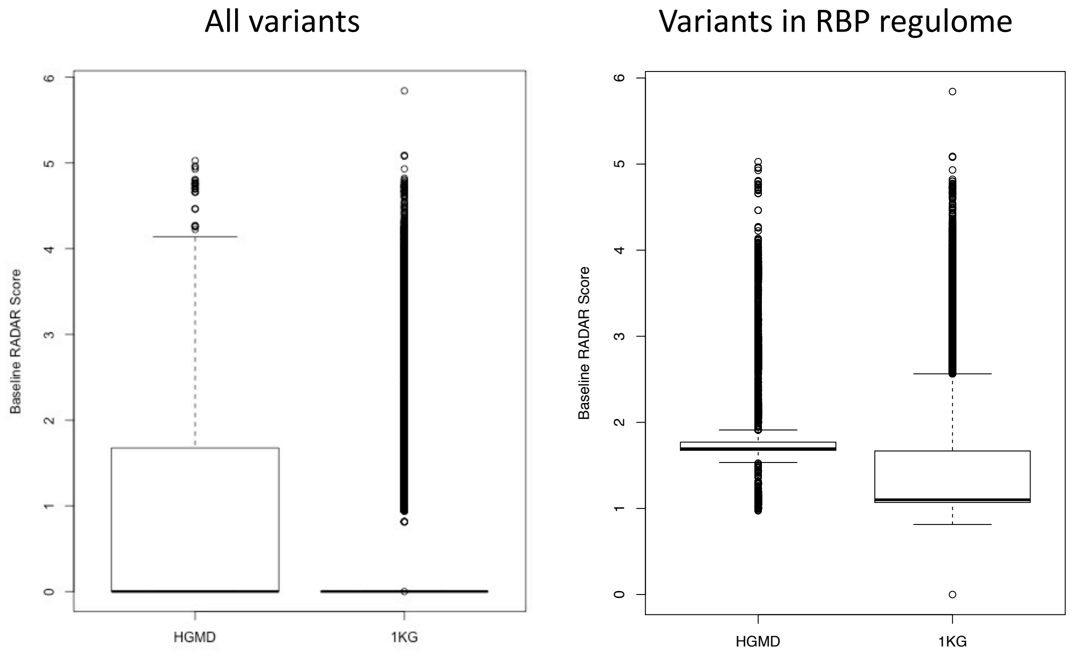

8 Comparison of RADAR to other methods

Below in Table S 3 we show the comparison of RADAR to FunSeq2 and also describe the relevance of each feature to variant prioritization on the RBP regulome.

Table S 3 Comparison of RADAR to Funseq2

Universal Features	Same as FunSeq2?	Relevance to RADAR
Cross-Population Conservation in eCLIP	N	Conservation of post-transcriptional regulome
Cross-Species Conservation	Y	Important for considering cross-species conservation.
Structural Conservation (Evofold)	N	RNA secondary structure
RBP Binding Hubs	N	Binding hubs are more conserved
RBP-gene associations	N	Gene expression changes caused by motif disruption
Motif Disruption	N	Disrupts binding of RBPs
**Tissue Specific Features**	**Same as FunSeq2?**	**Relevance to RADAR**
RBP Regulatory Potential	N	RBPs regulate gene networks
Differential Expression of Key Genes	N	DE is a hallmark of regulation
Mutational Recurrence	N	Recurrence in specific tissues demonstrate unique hotspots

9 Applying RADAR to somatic variants in cancer

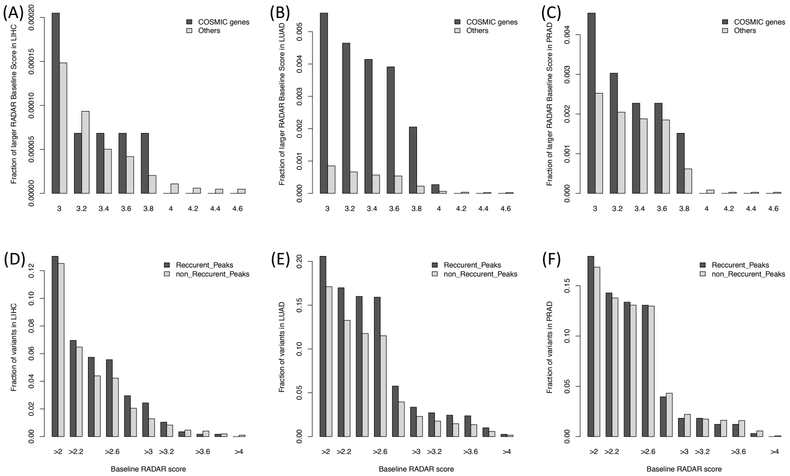

Figure S 10 RADAR score in somatic variants

The breast, liver, lung, and prostate cancer variants were downloaded from the paper by *Alexandrov et al[9]*. We first calculated the baseline RADAR scores on these four cancer types. We found that in most cancer types, COSMIC genes and recurrent RBP peaks are associated with more high impact variants. Results are shown in Figure S 10.

We also used expression profiles were downloaded from TCGA and the mutational variants as disease-specific features to prioritize breast cancer variants. Several more interesting examples from breast cancer were given in Figure S 11. We have listed the somatic variants that are only highlighted by RADAR in Additional file 1: T9 and Additional file 1: T10.

Figure S 11 Highlighted breast cancer variants in the 3' UTR region

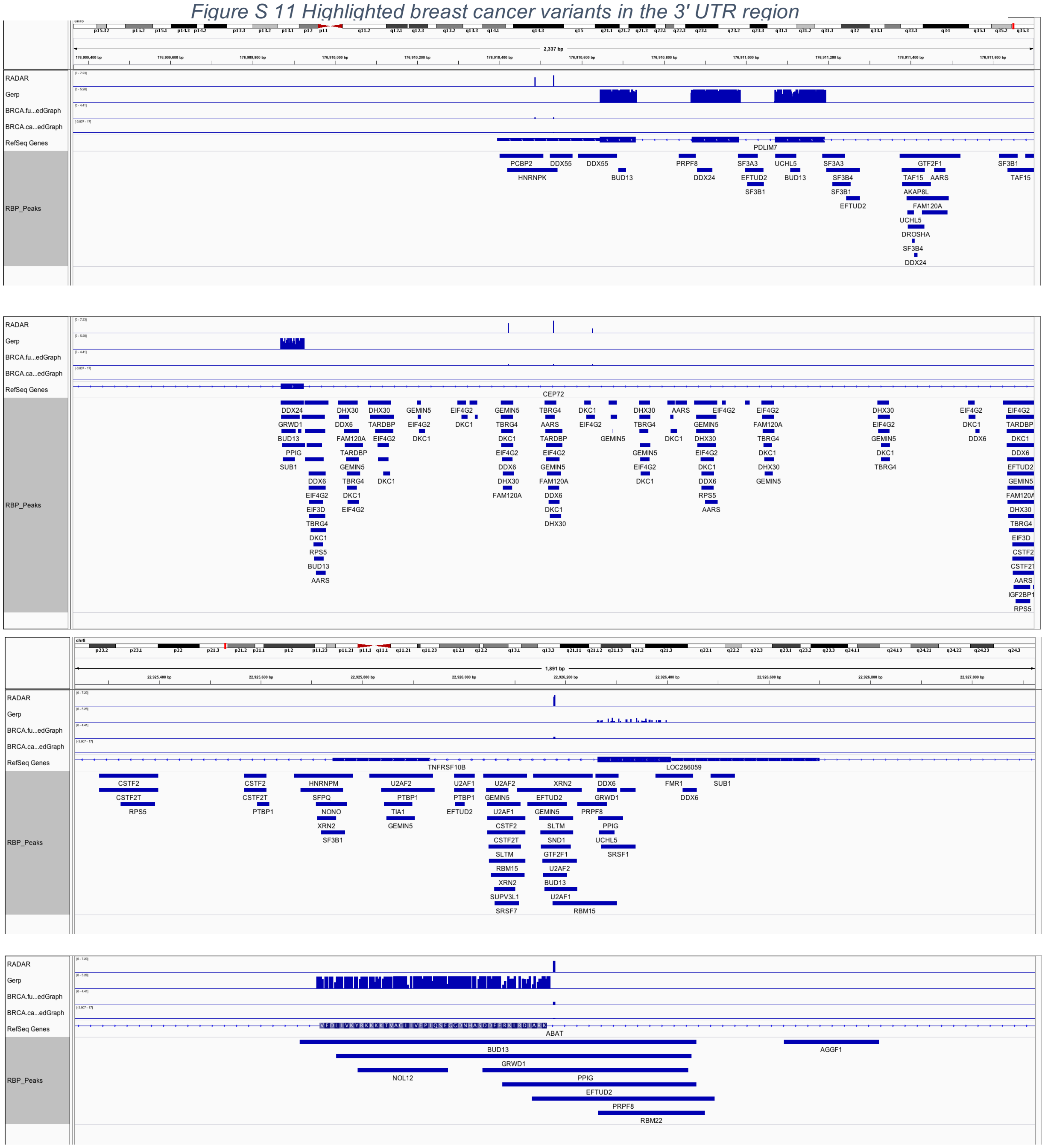

10 Relative Importance of RADAR Features

Figure S 12 Flowchart of Random forest analyses for RADAR variable importance

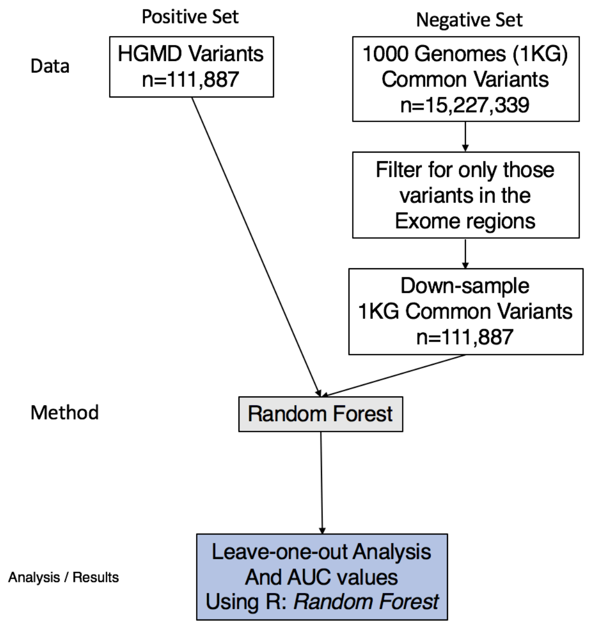

In both the random forest analyses (flowchart in Figure S 12), we take the HGMD germline disease variants as the positive set (n=111,887) and a random subset of the common germline variants from 1K Genomes as the negative set (n=111,887). In the first analysis, we run a random forest (# of trees = 500) using all features of the radar score to determine which feature is most important in predicting disease variants versus common variants. We find that the features such as cross population conservation in eCLIP and GERP are the most important. However, it should be noted that there is a slight bias due to GERP being naturally higher in the coding regions, where many of the HGMD variants lie. In addition, features like motif disruption are rare and therefore see smaller levels of AUC decrease when removed, even though they are important for outlier variants that have an extremely high RADAR score.

Below (Table S 4) is the table for the AUC results after leave-one-out analysis:

Table S 4 Summary of AUC values from leave-one-out analysis

**Left out Feature**	**AUC (after feature left out)**
FULL MODEL	0.889
Cross population cons. in eCLIP binding sites	0.766
GERP	0.735
Binding Hub	0.887
Motif Disruption	0.888
RNA Structure	0.887
RBP Gene Association	0.888

11 Using cell type specific information as validation

We used HepG2 and K562 specific data to build the RADAR model independently. Below are two examples illustrating the effect of cell type specific data on the RADAR score.

Example 1: Comparison of full RADAR scores on variants in common and differentially expressed genes in HepG2 and K562.

Here we show that somatic Liver cancer variants [9] falling in genes with both high expression in K562 and HepG2 (top 10% expression from total RNA-seq) demonstrate comparable scores when using matched cell type scoring schemes. Those variants falling in genes with high expression in HepG2 (top 10%) but low in K562 (FPKM<1) demonstrate scores that are much lower when using the K562 scoring scheme. The results are shown in Figure S 13.

Figure S 13. RADAR score comparison in tissue-specific and commonly expressed genes by separating K562 and HepG2 data

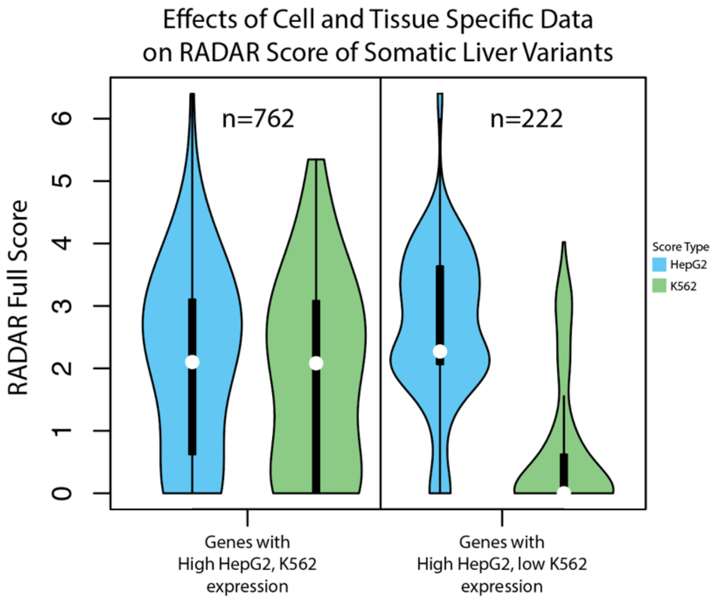

Example 2: scoring on somatic variants from tumor-specific and pancan driver genes

We compare the HepG2 and K562 scores for a set of Liver cancer variants available publicly from the Alexandrov et al paper [9] Here we observed that variants that fall in CTNNB1, a well-known cancer driver gene unique to liver cancer are scored much higher when using the HepG2 version of the score compared to the K562 version. As a control, we look at the scores of variants falling in TP53, a well-known cancer driver, but not specific to liver cancer. The results are shown in Figure S 14 below.

Figure S 14 RADAR score comparison in liver cancer specific and common tumor driver genes by separating K562 and HepG2 data

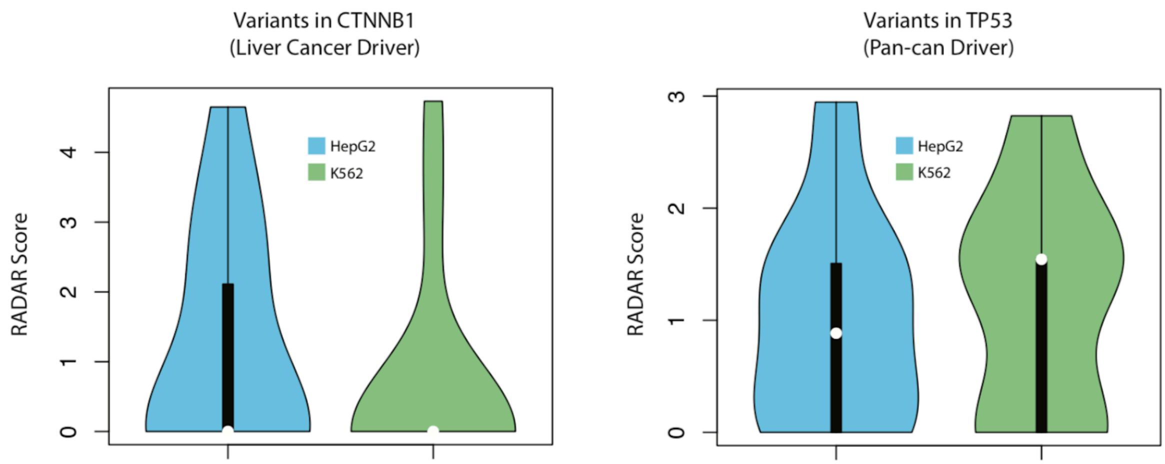

12 Comparison of CLIP methods

12.1 Comparison of eCLIP data to other RBP binding data

Figure S 15 Number of Peaks for Various RBP CLIP methods

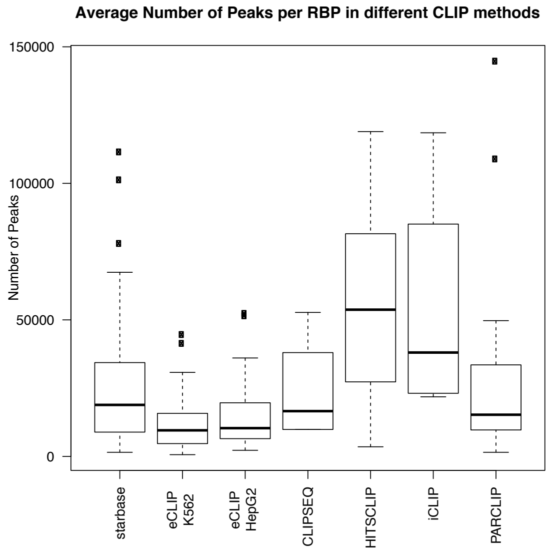

Figure S 15 shows the comparison of the number of peaks between different CLIP methods. The plot serves as a quality check of the eCLIP data, indicative of a more stringent cutoff to ensure high quality peaks. In addition, the number of peaks across different cell types (K562 and HepG2)

is similar within eCLIP data. The variance is also demonstratively lower in the eCLIP data as compared to other methods.

13 Cross-population and species conservation and GC bias

13.1 ***Inference of cross-population conservation of RBP binding sites***

We tried to infer the cross-population conservation level of the RBP binding sites from polymorphism data in large sequencing cohorts like the 1,000 Genomes Project. Specifically, for each RBP we divided all the binding peaks into coding and noncoding regions separately and then calculated the number of common (n_c) and rare variants (n_r) in these two categories. Then a one-sided binomial test of n_c, n_r, vs. the genome background f was calculated to evaluate the enrichment of rare variants.

However, in our analysis we found that GC content might be a potential bias in such calculation. As in Figure S 16, the background rare variant percentage f demonstrates noticeable changes with GC percentage. One possible explanation is that GC content usually affects read coverage in high-throughput sequencing experiments, which is a sensitive parameter in the downstream variant calling process. Therefore, to remove such bias, we calculated the GC adjusted background rare variant percentage by dividing the coding/noncoding regions into 500bp bins, and grouping these bins at GC resolution of 0.02. For each RBP, when calculating the background, we only select the bins with closest GC percentage. The comparison of foreground and background rare variant percentage for every RBP in coding and noncoding regions are given in Figure S 17.

Figure S 16. Background Rare variant percentage vs. GC

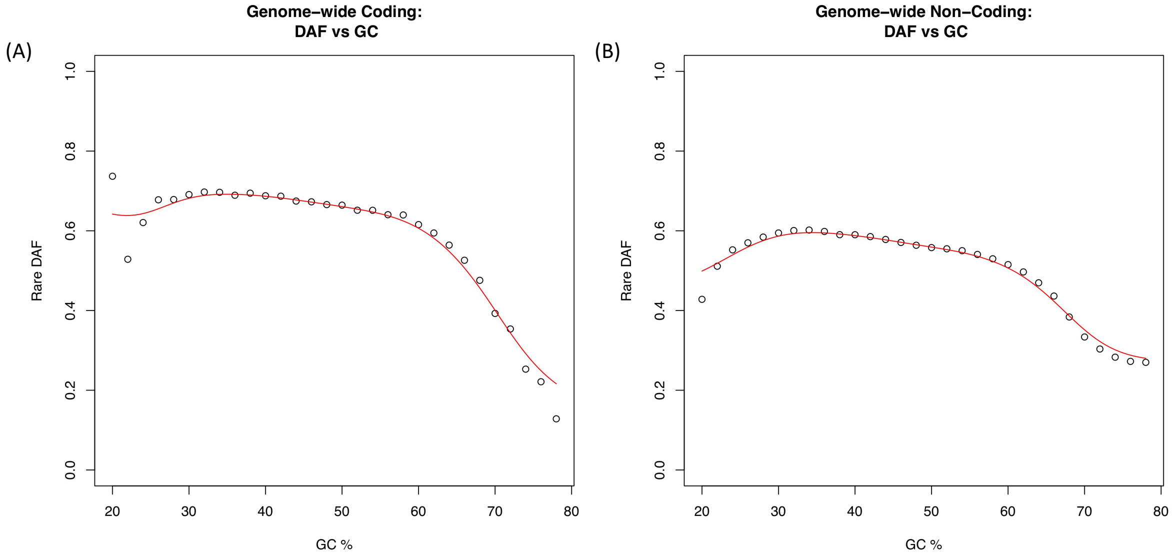

For some RBPs, if there are no coding/noncoding rare/common variants in their binding sites, the f value for binomial test will be missing. We provided the full raw calculation of GC corrected rare variant enrichment for each RBP in Additional file 1: T2.

Figure S 17 Rare variant enrichment after GC correction in coding and noncoding regions respectively. The dashed blue/red line is the genome average without GC correction for coding and noncoding regions, and the solid blue/red line is the background after GC correction. Blue/Red star on top of each bar indicate significantly enriched in rare variants after GC correction in one-sided binominal test against the coding/noncoding average.

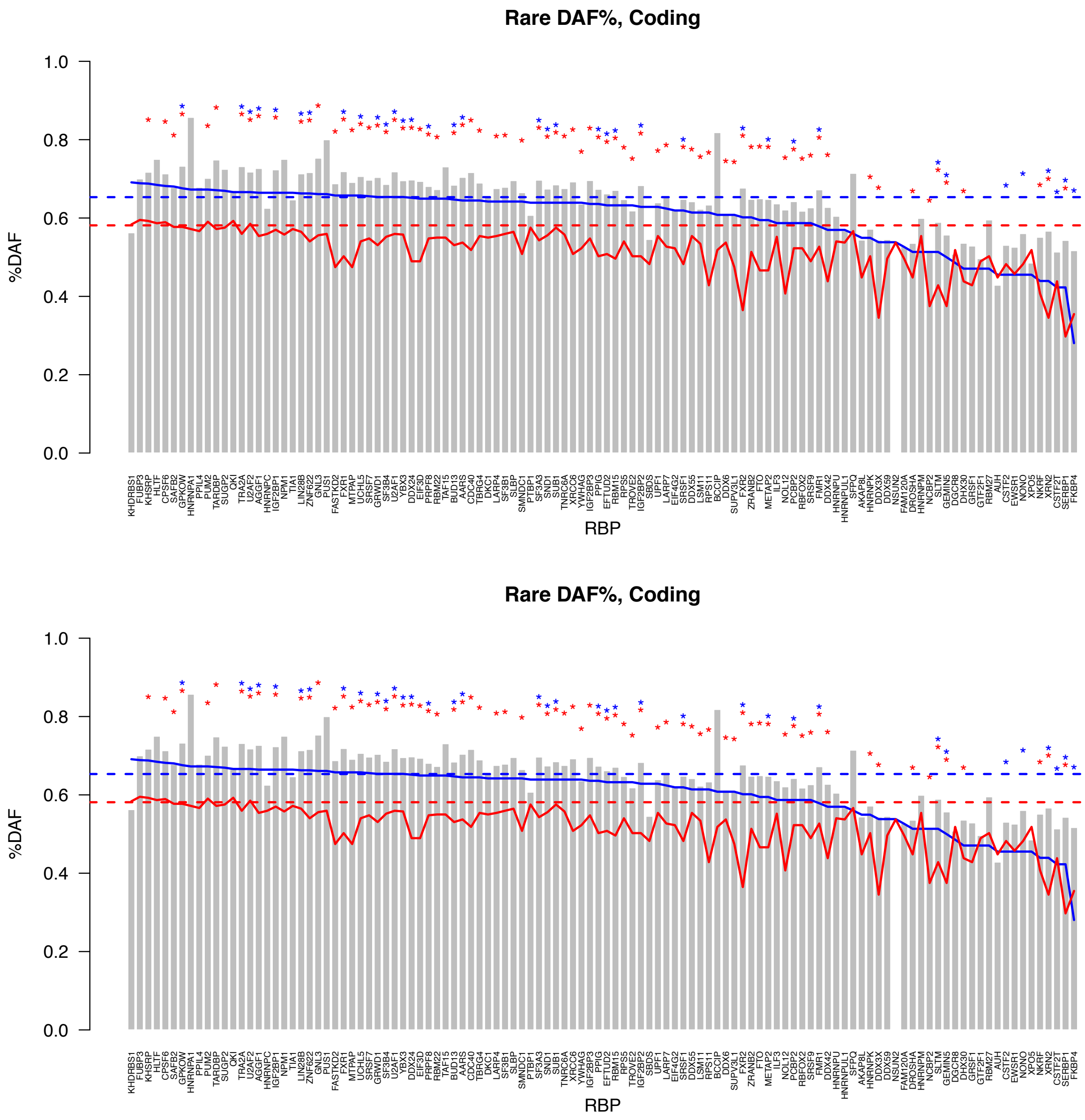

13.2 ***Inference of cross-species conservation of RBP binding sites***

PhastCons conservation scores were downloaded from UCSC genome browser. For each annotation category (coding exons, 3’UTR, 5’UTR, nearby introns), we separate the annotation into regions covered by RBP peak and those not covered. After deduplication and merging of the bed files, we then calculated the average PhastCons score in each region using bigWigAverageOverBed (downloaded from UCSC genome browser). Then the boxplots of peak vs. nonpeak regions were given in Figure 2 of the main manuscript.

In addition the GERP score sigmoid transformation is given as the equation below:

$$S_{GERP} = 0.62 \times \frac{1}{1 + e^{-40(GERP-1.85)}}$$

14 Motif analysis

In our RADAR framework, we incorporated two sources of motifs: (1) motifs from RNA Bind-n-Seq experiments[10]; (2) *de novo* discoveries from RBP peaks by DREME [11]. For each variant, we used the changes of PWM scores to quantify the binding affinity alterations. If one variant breaks more than one PWM, RADAR will choose the maximum score for it.

14.1 ***Motifs from RNA Bind-n-Seq experiments***

It has been reported that many of the RBPs’ binding events *in vivo* can be captured by binding preferences *in vitro*. Hence, we utilized an in vitro RNA binding assay, RNA Bind-N-Seq[10] to characterize sequence and structural specificities of RBPs. We used RBNS motifs from 78 human RBPs to prioritize germline and somatic variants that could potentially disrupt an RNA-binding domain.

Briefly, we called on RBNS motifs based on an enrichment Z-score cutoff of 3. Some RBPs had up to four motifs, which ranged from 5-mer to 9-mers. In total, there are 17 RBPs overlapped with eCLIP RBPs, which are listed in Table S 5 below. We treated all RBNS motifs independently from eCLIP-based *de novo* motifs.

Table S 5 List of RBPs that have both eCLIP and Bind-n-Seq experiments

	RBP Name	RBNS motif
1	EIF4G2	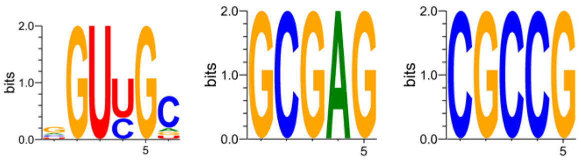

2	EWSR1	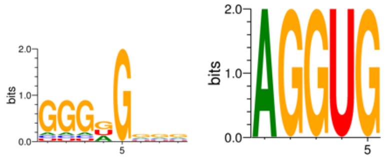
3	FUBP3	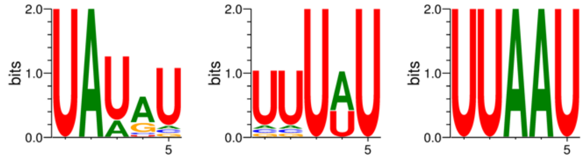
4	HNRNPC	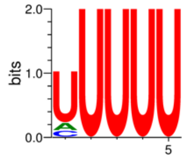
5	HNRNPK	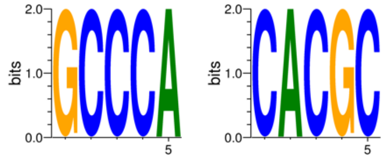
6	IGF2BP1	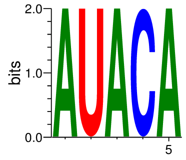
7	IGF2BP2	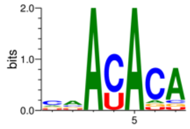
8	KHSRP	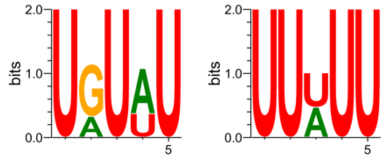
9	PCBP2	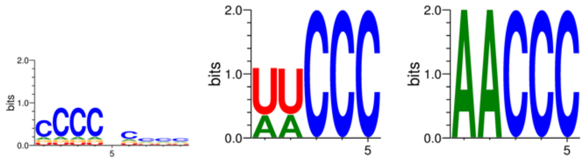
10	RBFOX2	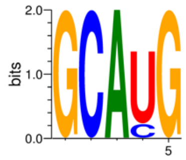

11	RBM22	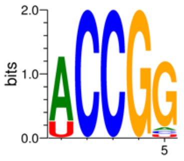
12	SFPQ	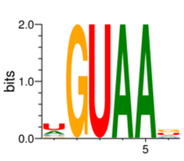 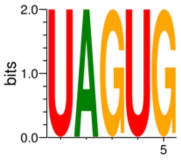
13	SRSF9	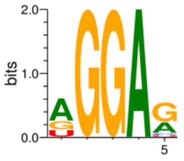 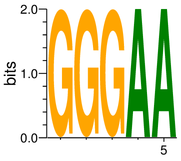
14	TAF15	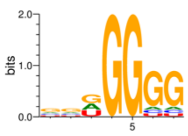 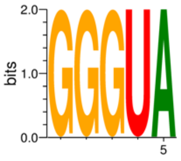
15	TARDBP	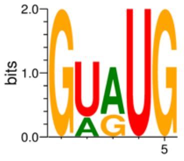 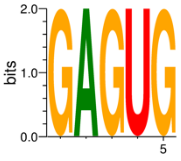
16	TIA1	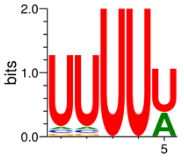
17	TRA2A	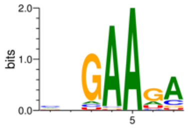

14.2 Motifs from de novo discovery

We collected the binding peaks for each RBP after blacklist removal. For any peak that is less than 150 bp in length, we extended it to 150 bp from both sides. For those longer than 150bp, we kept the original peak length. We then extracted sequence information from hg19 and performed *de novo* motif discovery DREME[11] with default settings (Version 4.12.0, http://meme-suite.org/tools/dreme).

14.3 Motif disruption calculation using MotifTools

We used D-score defined in MotifTools (https://github.com/hoondy/MotifTools) to evaluate the binding affinity alterations introduced by a variant. We only considered positive D-scores, which denote a variant that decreases the likelihood that a protein will bind the motif (motif-break). For variants that affect multiple RBP binding profiles, we used the max score over all D-scores.

15 Downloading and Using the RADAR software

We include a downloadable ZIP file at http://radar.gersteinlab.org/#!page-downloads which contains the RADAR source code (radar.py) and a directory containing all data files needed by RADAR (resources/). Individual files are listed below for download, and include descriptions of each file. This website also provides software documentation, usage information, performance benchmarks, and test examples. We also provided a web version of the software that can be used to run RADAR directly through the site. A variant file (BED format) can be uploaded, with the option to select any tissue-specific features. The output contains each score feature as well as the full RADAR score.

RADAR can also be run from the command line after unzipping radar.zip and downloading the necessary dependencies (Python, BEDTools and pybedtools). Users can run the software using the following command:

```
python radar.py -b [BED file containing variants to be scored] -o
[output directory] –a [assembly] -c [cancer type] [-kg -mr -rp]
```

```
[yf95@farnam1 ~]$ python
Python 2.7.13 (default, Jun  1 2017, 16:52:45)
[GCC 5.4.0] on linux2
Type "help", "copyright", "credits" or "license" for more information.
>>> import pybedtools
>>>
```

The –a argument may optionally be provided to specify which genome assembly to use for scoring. By default, RADAR uses hg19, but also supports GRCh38. The -kg, -mr and -rp are optional parameters that are used to indicate whether tissue-specific scores (key genes, mutation recurrence, and RBP regulation power) should be computed. These options require specifying a TCGA cancer abbreviation. Note that currently, tissue-specific scores are only available for hg19. After running the software, the output scores can be found in

```
[input BED file name].radar_out.bed
```

The RADAR source code can be found at https://github.com/gersteinlab/RADAR. Included below is a step-by-step walkthrough of using RADAR to score the Alexandrov *et al* breast cancer variants.

Dependencies for RADAR include BEDTools (which can be found at http://bedtools.readthedocs.io/en/latest/content/installation.html), Python (which can be found at https://www.python.org/downloads/), and pybedtools

```
[yf95@farnam1 ~]$ bedtools
bedtools is a powerful toolset for genome arithmetic.

Version:   v2.27.1
About:     developed in the quinlanlab.org and by many contributors worldwide.
Docs:      http://bedtools.readthedocs.io/
Code:      https://github.com/arq5x/bedtools2
Mail:      https://groups.google.com/forum/#!forum/bedtools-discuss

Usage:     bedtools <subcommand> [options]
```

(http://daler.github.io/pybedtools/main.html). Our RADAR software is compatible for both Python version 2 and 3. For more details please see radar.gersteinlab.org/#!page-downloads.

The RADAR package can be downloaded at http://radar.gersteinlab.org/#!page-downloads. The unzipped file includes a radar directory contains python executable script and a resources directory containing all data files needed by the RADAR script to produce scores.

The example file (Breast.bed) is downloadable at http://radar.gersteinlab.org/#!page-example. Other examples with high scoring RADAR variants can also be found at the page.

```
[yf95@farnam1 example]$ head Breast.bed
chr1    13506   13507   G       A       TCGA-EW-A1OZ-01A-11D-A142-09
chr1    14841   14842   G       T       PD5935a
chr1    16995   16996   T       C       PD7201a
chr1    17764   17765   G       A       PD5935a
chr1    17764   17765   G       A       PD7216a
chr1    28587   28588   G       T       PD4962a
chr1    30527   30528   C       T       PD5935a
chr1    61396   61397   G       A       PD4967a
chr1    69522   69523   G       T       TCGA-BH-A0BP-01A-11D-A10Y-09
chr1    83442   83443   C       T       PD4072a
```

We run the software on the command line as follows:

```
python radar.py -b ../Breast.bed -o .. -c BRCA -kg -mr -rp
```

```
[yf95@farnam1 radar]$ python radar.py -b ../Breast.bed -o .. -c BRCA -kg -mr -rp
[yf95@farnam1 radar]$ ls ..
Breast.bed  Breast.radar_out.bed  radar  radar.zip
```

RADAR has generated the output file, Breast.radar_out.bed, which contains the list of scored variants. A head of the output file is shown below (note that the header takes up one line in the file, but is broken onto two lines in this screenshot):

```
[yf95@farnam1 radar]$ head ../Breast.radar_out.bed
chr	start	stop	ref	alt	cross_species_conservation	RBP_binding_hub GERP	Evofold motif_disruption	RBP_gene_association
total_universal	key_genes	mutation_recurrence	RBP_regulation_power	total_tissue_specific	total_score
chr1	13506	13507	G	A	0	0	0	0	0	0	0	0	0	0	0	0
chr1	14841	14842	G	T	0	0	0	0	0	0	0	0	0	0	0	0
chr1	16995	16996	T	C	0	0	0	0	0	0	0	0	0	0	0	0
chr1	17764	17765	G	A	0	0	0	0	0	0	0	0	0	0	0	0
chr1	28587	28588	G	T	0	0	0	0	0	0	0	0	0	0	0	0
chr1	30527	30528	C	T	0	0	0	0	0	0	0	0	0	0	0	0
chr1	61396	61397	G	A	0	0	0	0	0	0	0	0	0	0	0	0
chr1	69522	69523	G	T	0	0	0	0	0	0	0	0	0	0	0	0
chr1	83442	83443 _	C	T	0	0	0	0	0	0	0	0	0	0	0	0
```

Please note that we also provide a version of the RADAR score for the hg38 assembly. The fully computed score on the RBP regulome can be found at http://radar.gersteinlab.org/#!page-downloads.

16 References

1. Lovci MT, Ghanem D, Marr H, Arnold J, Gee S, Parra M, Liang TY, Stark TJ, Gehman LT, Hoon S, et al: **Rbfox proteins regulate alternative mRNA splicing through evolutionarily conserved RNA bridges.** *Nat Struct Mol Biol* 2013, **20:**1434-1442.
2. Ye J, Beetz N, O'Keeffe S, Tapia JC, Macpherson L, Chen WV, Bassel-Duby R, Olson EN, Maniatis T: **hnRNP U protein is required for normal pre-mRNA splicing and postnatal heart development and function.** *Proc Natl Acad Sci U S A* 2015, **112:**E3020-3029.
3. van Roon AM, Oubridge C, Obayashi E, Sposito B, Newman AJ, Seraphin B, Nagai K: **Crystal structure of U2 snRNP SF3b components: Hsh49p in complex with Cus1p-binding domain.** *RNA* 2017, **23:**968-981.
4. Lin PC, Xu RM: **Structure and assembly of the SF3a splicing factor complex of U2 snRNP.** *EMBO J* 2012, **31:**1579-1590.
5. Obeng EA, Ebert BL: **Charting the "Splice" Routes to MDS.** *Cancer Cell* 2015, **27:**607-609.
6. Rousseau F, Labelle Y, Bussieres J, Lindsay C: **The fragile x mental retardation syndrome 20 years after the FMR1 gene discovery: an expanding universe of knowledge.** *Clin Biochem Rev* 2011, **32:**135-162.
7. Crawford DC, Acuna JM, Sherman SL: **FMR1 and the fragile X syndrome: human genome epidemiology review.** *Genet Med* 2001, **3:**359-371.

8. Love MI, Huber W, Anders S: **Moderated estimation of fold change and dispersion for RNA-seq data with DESeq2.** *Genome Biol* 2014, **15:**550.
9. Alexandrov LB, Nik-Zainal S, Wedge DC, Aparicio SA, Behjati S, Biankin AV, Bignell GR, Bolli N, Borg A, Borresen-Dale AL, et al: **Signatures of mutational processes in human cancer.** *Nature* 2013, **500:**415-421.
10. Lambert N, Robertson A, Jangi M, McGeary S, Sharp PA, Burge CB: **RNA Bind-n-Seq: quantitative assessment of the sequence and structural binding specificity of RNA binding proteins.** *Mol Cell* 2014, **54:**887-900.
11. Bailey TL: **DREME: motif discovery in transcription factor ChIP-seq data.** *Bioinformatics* 2011, **27:**1653-1659.
